# Geometry Effects of Axisymmetric Flow-Focusing Microchannels for Single Cell Encapsulation

**DOI:** 10.3390/ma12172811

**Published:** 2019-09-02

**Authors:** Mohammad Nooranidoost, Ranganathan Kumar

**Affiliations:** Department of Mechanical and Aerospace Engineering, University of Central Florida, Orlando, FL 32816, USA

**Keywords:** flow-focusing, cell encapsulation, microfluidics, front-tracking method

## Abstract

Cell microencapsulation is a promising technique to protect living cells in biomedical applications. Microfluidic devices can be utilized to control the production of high-throughput cell-laden droplets. This paper demonstrates the effects of flow-focusing geometry on the droplet size, frequency of droplet generation, and number of cells per droplet. Orifice radius, orifice length, and nozzle-to-orifice distance can significantly influence the flow-field and manipulate droplet formation. This paper analyzes these geometry effects using a numerical front-tracking method for the three fluid phases. It is found that as the orifice radius increases, the drop size and the number of cells in the droplet increase. For a short orifice radius, increasing the orifice length results in the generation of smaller droplets at higher frequency and fewer cells per droplet. On the other hand, for a longer orifice, droplet production is invariant with respect to orifice length. It is also found that shorter distances between the nozzle and the orifice lead to a more controlled and uniform production of droplets. When the nozzle-to-orifice length is increased, the droplet formation becomes non-uniform and unpredictable. Probability charts are plotted with respect to the orifice length and orifice radius, which show that a greater than 50% probability of single cell encapsulation can be achieved consistently.

## 1. Introduction

Flow-focusing microencapsulation is a technique to encapsulate living cells in order to protect them in a safe environment and avoid any cell damage and rupture. In this technique, a shell of encapsulating fluid covers the cell and isolates it from the ambient fluid. Thus, the cell remains alive and viable and can be used for therapeutic and non-therapeutic applications. This shell provides an environment similar to what the cells experience in vitro [1]. Cell microencapsulation is a promising strategy in tissue engineering [2], drug delivery [3,4], bio-printing systems [5], cancer treatments [6], and food industries [7]. It helps therapeutic procedures by trapping living single cells such as fibroblasts, myoblasts, or stem cells within a semi-permeable membrane. This membrane allows the influx of nutrients and oxygen, as well as the efflux of metabolic waste products [8]. Various techniques such as droplet microfluidics have been utilized to produce cell-laden droplets. The geometry of these microfluidic devices can play an important role in single cell encapsulation. Thus, understanding the effects of microchannel geometry on the production of cell-laden droplets is of interest and provides researchers an insight into the physics of the encapsulation process and in the design of an appropriate microfluidic device for single cell encapsulation.

In recent years, microfluidics has provided a successful platform for the production of cell-laden droplets with high efficiency. The probability of cell-laden droplets, as well as the capability to produce high-throughput uniform droplets are the main characteristics of a highly-efficient single cell encapsulation system [9]. Moon et al.’s statistical study [10] showed that the initial random distribution of cells in a dispersed fluid follows a Poisson distribution in terms of the numbers of cells in a droplet. However, the probability of having single cells per droplet can be improved by evenly spaced [11] and closed packed [12] ordering of the cells within the channel. As discussed later, the cells in the current study are also initiated with an evenly-spaced ordering at the inlet to increase the chance of single cell encapsulation. Digital microfluidics or the droplet-based microfluidic technique can be employed to generate droplets in various types of microfluidic devices for cell encapsulation. T-junction, cross-junction, and flow-focusing are the most common methods used in microencapsulation of living cells [13]. These devices possess various design features that can impact flow dynamics in the microchannels significantly. In these microchannel designs, the most effective area is the junction where the continuous and dispersed fluids intersect and pinch off due to the pressure and the stress applied at the interface between the two fluids. The flow pattern in these micro devices depends on the flow parameters and dimensions of the junction area, where droplets form and cell encapsulation occurs.

In T-junction devices, the continuous fluid applies pressure on the dispersed fluid when the two fluids meet at the intersection. The upstream pressure acts against the stabilizing interfacial tension force, and when the pressure becomes dominant, it pushes the protruding droplet downstream of the microchannel. The interplay between these forces results in the formation of a thread, which leads to detachment and eventual pinch-off of the droplet [14,15]. Researchers have successfully generated hydrogel microbeads [16,17,18] and polymeric microparticles [19] in T-junction devices. Depending on the cell population in the dispersed fluid, the droplet may not contain any cell or contain one or multiple cells. These devices have the advantage of simplicity in fabrication and are easy to use. On the other hand, the shear stresses in the junction area may become too high and can easily damage cells. Therefore, the flow conditions need to be kept in a certain range to avoid cell damage. To facilitate this, the frequency of droplet generation in these devices is usually limited to several hundred Hertz [13].

Cross-junction microchannels are another type that is commonly used to generate droplets for cell encapsulation [11,20,21]. In these devices, the continuous and the dispersed fluid meet at the junction where high velocity and flow focusing can occur. The evolution of the interface is smooth, and the mechanical stresses applied on the cells during droplet detachment are less than those in T-junction techniques, which leads to smaller deformation of the cell. Therefore, it is possible to achieve high-throughput single cell encapsulation with maximum cell viability [13].

In flow-focusing geometries such as the ones analyzed in this paper, both dispersed and continuous fluids enter the microchannel in parallel. The two immiscible fluids meet while flowing in the same direction through an orifice. At the orifice, both flows focus in a narrow region where high velocities enable the formation of droplets with very high frequencies [22,23,24,25]. In flow-focusing devices, the orifice raises the local pressure around the droplet thread. The driving pressure force may overcome the resisting forces of surface tension and viscosity to pinch-off the droplet [22]. This process leads to the formation of a compound droplet, which flows downstream [23,26,27]. The axisymmetric flow-focusing device also has the advantage of focusing the dispersed fluid to a narrow circular channel in the orifice region, which orients the cells at the centerline, a condition that is favorable for single cell encapsulation [23]. The parallel axisymmetric flow-focusing configuration has two main advantages. The continuous fluid does not contact the outer channel wall, which mitigates wetting issues and can favorably lead to a stable droplet pinch-off. The second advantage is that these devices can be operated at both high and low pressures without any fluid leak at the seals [28]. However, such fabrication can be complicated as shown by Takeuchi et al. [28], who fabricated an axisymmetric flow-focusing device using glass capillaries and an insulated optical fiber. They cut the glass capillaries, inserted the optical fiber in the middle of capillaries, and removed the fibers so that the optical fiber tube could serve as the orifice.

The effects of geometry on droplet formation in these microfluidic devices have been mainly studied numerically. Such simulations allow researchers to predict new physics and the parameter space in which favorable patterns of droplet formation and single cell encapsulation emerge, without the need to fabricate various devices. Wehking et al.’s numerical study [15] on a T-junction showed the noticeable role of geometry on the transition between different regimes and the change in droplet size/droplet production frequency by increasing the height-to-width ratio of the channel. Garstecki et al. [29] also observed that the width of channels in T-junctions can control the shear stress exerted on the interface between fluids, which distorts the droplet and can characterize different droplet formation regimes. Liu et al.’s three-dimensional numerical study [30] in low capillary numbers show that the width ratio of main and lateral channels in cross-junction configurations can influence the droplet formation pattern. The pinch-off process becomes more difficult, and droplet size increases as the width ratio of the main-to-lateral channel increases. Gupta et al. [31] studied the effects of geometry on droplet formation in 2D flow-focusing configurations. Their simulations revealed that droplet production at low capillary numbers is due to the pressure build-up in the continuous phase and the interfacial force and squeezing of the interface. The droplet size grows exponentially by shortening the orifice length, while for a long orifice, it approaches a constant value. The droplet size also increases as the orifice width and the distance of the orifice from the inlet increase. Wu et al. [32] compared droplet production in co-flowing and axisymmetric flow-focusing devices. Their numerical study showed the important role of the orifice in the flow-focusing geometry by inducing hydrodynamic focusing effect. The droplet formation was significantly affected by the orifice radius, while it was insensitive to the orifice length. Recently, Yu et al.’s work [33] on cross-junction devices revealed that the variation in the junction angle changes the hydrodynamic characteristics in and around the junction region, manipulates the interface evolution occurring in that region, and therefore changes the formation of droplets.

In spite of the many geometrical studies on the droplet formation in microchannels, to the best of our knowledge, there is no work on the geometrical effects on cell encapsulation in three-phase systems. This work focuses on the effects of geometry on droplet formation and cell encapsulation in an axisymmetric flow-focusing device. The three main geometrical parameters that can characterize the droplet formation pattern and identify the modes of cell encapsulation are: (1) orifice radius, (2) orifice length, and (3) nozzle-to-orifice distance. A detailed study was conducted to investigate the role of these geometrical parameters on droplet size, the frequency of cell encapsulation, and the cell population inside the encapsulating droplet. The axisymmetric microchannel device is state-of-the-art, as shown by Takeuchi et al. [28], and there is a dearth of information on this type of device. Therefore, the simulations performed in this paper for various orifice geometry parameters such as the orifice aspect ratio and the orifice placement with respect to the inner tube can maximize the probability of single cell encapsulation and will be useful in microfluidic design. In addition, the information provided in this paper can guide researchers to fabricate the device with their desired dimensions for improved microencapsulation.

## 2. Cell Encapsulation Process

### 2.1. Problem Statement

**Figure 1:**
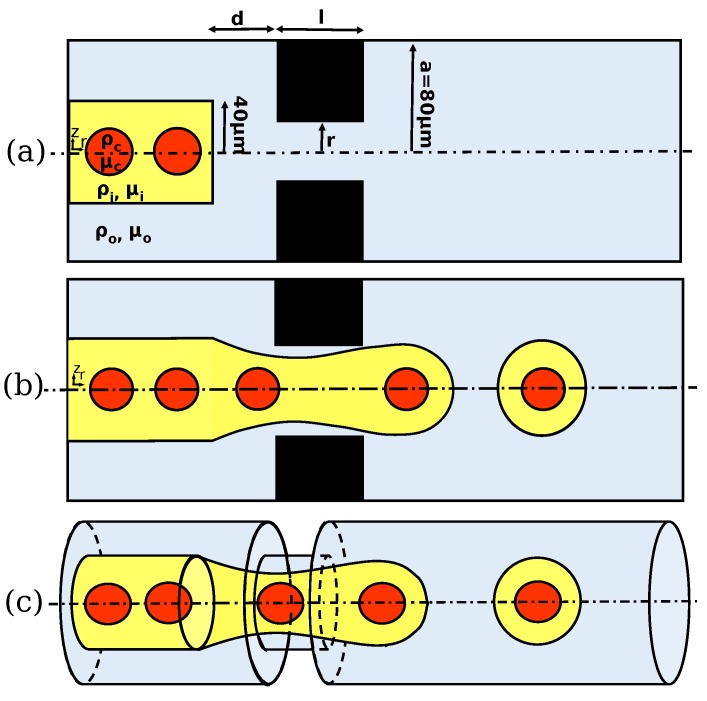
The schematic illustration of the flow-focusing geometry and encapsulation process: (**a**) initial position; (**b**) after pinch-off of the compound droplet; (**c**) 3D illustration of the encapsulation process.

The encapsulation of single cells is shown schematically in Figure 1. The axisymmetric flow-focusing geometry consists of a main microchannel with a circular cross-section (the radius of the main microchannel is 80 μm), a smaller concentric subchannel (the radius of the subchannel is 40 μm) at the entrance, and an orifice downstream of the main channel. The cells, the dispersed fluid (inner fluid), and the continuous fluid (outer fluid) are shown in red, yellow, and blue colors, respectively (Figure 1). The interface between dispersed and continuous fluid is initially flat at the exit of the inner subchannel, and twenty uniform deformable circular cells (the radius of the cell is 10 μm) are initiated with evenly-spaced ordering (the center-to-center distance between cells is 30 μm) inside the inner subchannel (Figure 1a). The dispersed and continuous fluids enter the microchannel in the same direction from the inner subchannel and the annulus, respectively. They flow in parallel flow streams, and when they reach the orifice region downstream of the channel, they focus to a narrower cross-section. The evolution of the interface is shown schematically in Figure 1b. As the dispersed fluid begins to penetrate the continuous fluid, the cell loads into a forming droplet. Viscous stresses induced by the continuous fluid and interfacial forces shape the evolution of the interface. Eventually, necking occurs in the orifice region, and the compound droplet forms downstream of the microchannel (Figure 1b). The flow is assumed to be incompressible and axisymmetric. Fully-developed velocity profiles are imposed in the inlet of both the inner and annular microchannels. The pressure is kept constant at the outlet, and the no-slip boundary condition is used at the wall of the tube.

In all of the simulations presented in this work, the average velocity of both the inner ($V_{i}$) and outer fluids ($V_{o}$) was set to be 0.01 m/s in the inlet of the inner and annulus tube, respectively. Interfacial tension of shell fluid/cell and continuous fluid/shell fluid were set to be the same as σ = 0.02 N/m, and the viscosity of all three fluids was fixed as $\mu_{c}=\mu_{i}=\mu_{o}=0.002$ Pa·s. We define the capillary number, which represents the relative effect of viscous forces on the interfacial forces acting across the interface of the continuous fluid/shell fluid. The capillary numbers for the inner and outer fluids are defined as $Ca_{i}=\mu_{i}V_{i}/\sigma$ and $Ca_{o}=\mu_{o}V_{o}/\sigma$, respectively, and are fixed in all the simulations as $Ca_{i}=Ca_{o}=10^{-3}$, unless specified otherwise. Note that due to the reduction of the cross-section area, velocity in the orifice region was one order of magnitude larger than what it was at the inlet. Hence, the local effective capillary number was one order of magnitude larger than the reported reference capillary number.

Fluid and flow parameters, as well as the geometry of the microchannel can play significant roles in the droplet formation process, which characterizes a particular mode of cell encapsulation. They can control the droplet size, uniformity of the droplets, frequency of droplet production, and population of cells inside the droplets. The effects of fluid and flow properties were studied in [23], and the aim of this work is to study the effects of microchannel geometry on the cell encapsulation process. Here, the three characteristic lengths that play an important role in the encapsulation of cells are the orifice radius (r), orifice length (l), and nozzle-to-orifice distance (d). These parameters are nondimensionalized with the radius of the main microchannel (a=80 μm) and are presented as:

$$\begin{array}{c} r^{*}=r/a,\;l^{*}=l/a,\;d^{*}=d/a. \end{array}$$

We defined a base case as $r^{*}=0.25$, $l^{*}=0.5$, and $d^{*}=0.75$, and studied the sole effects of geometrical parameters by varying them while keeping the other parameters fixed. Simulations were performed to investigate the effects of these three geometrical parameters on droplet size, frequency of droplet generation, and probability of cell population per droplet. The droplet size was represented by the average volume of the generated droplets, either with or without cells. It was normalized using the initial cell volume ($V_{cell}=4.19\times 10^{-6}$ μL). Furthermore, the probability of cell population per droplet was calculated as the ratio of the number of droplets with no cells, with one cell, or multiple cells to the total generated droplets. Note that the probability was calculated after the flow reached a steady state condition.

### 2.2. Physics of Encapsulation in Axisymmetric Flow-Focusing Microchannels

**Figure 2:**
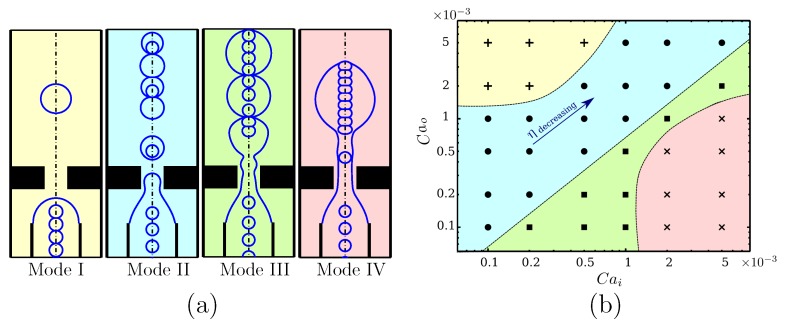
(**a**) Different modes of cell encapsulation. (**b**) Phase diagram of different modes. Color patches represent different cell encapsulation modes: yellow: Mode I, mostly empty droplets with no cells inside; blue: Mode II, at most one cell per droplet; green: Mode III, at least one cell per droplet; pink: Mode IV, no breakup to form cell-encapsulated droplets; η = success rate of encapsulation. Reprinted with permission from Nooranidoost et al. [23]. Copyright (2019) Springer.

Fluid and flow properties and the geometrical setting of the flow-focusing device play important roles in flow patterns and the regime of droplet generation. Nooranidoost et al. [23] numerically studied the effects of fluid and flow properties on cell encapsulation. In particular, they investigated the effects of the viscosity of the inner fluid and flow rates of both inner and outer fluids on the modes of encapsulation, droplet size, frequency of cell loading, frequency of droplet generation, and population of cells inside the droplet. They observed four distinct modes of encapsulation: mostly empty droplet with no cells inside (Mode I); at most one cell per droplet, also known as single cell encapsulation (Mode II); at least one cell per droplet (Mode III); and no break up to form cell-encapsulated droplets (Mode IV) (Figure 2a). In therapeutic applications, single cell encapsulation is the desired mode, since cell-encapsulated droplets can occur without coalescence of cells. Their parametric study also revealed that for a fixed geometry of the flow-focusing device, the viscosity of the inner fluid and flow rates of the inner and outer fluids can significantly change the mode of encapsulation. The flow rates of the inner and outer fluids can be adjusted to reach the desired mode of encapsulation. Figure 2b shows the phase diagram of different encapsulation modes for a range of inner and outer capillary numbers at fixed viscosities $\mu_{i}=\mu_{o}=0.002$ Pa·s. Modes I, II, III, and IV are shown in yellow, blue, green, and pink patches, respectively, and the approximated trend lines show the border between different modes (Figure 2b). Figure 2b also shows that the ratio of capillary numbers, i.e., $Ca_{i}/Ca_{o}$, may be a possible parameter to identify the modes of encapsulation. For $Ca_{i}/Ca_{o}\ll O\left(1\right)$, a high flow rate of the outer fluid hinders the cell flow to form a cell-laden droplet, resulting in the production of mostly empty droplets (Mode I; yellow patch). For higher ratios of capillary numbers, i.e., $Ca_{i}/Ca_{o}≃O\left(1\right)$, droplets are generated with a frequency comparable to the frequency of cells entering the channel. Therefore, cells have a probability of at least 10% to produce cell-laden droplets. However, there is also some possibility of empty droplets with no cells. Thus, the population of cells inside the droplet depends highly on the physical conditions, which can be adjusted for the desired cell population (Mode II; blue patch and Mode III; green patch). Finally, for high capillary number ratios $Ca_{i}/Ca_{o}\gg O\left(1\right)$, the high flow rate of the inner fluid may not allow the forming droplet to break up encapsulating cells (Mode IV; pink patch). This is illustrated in Figure 2b in Mode IV, where multiple cells form inside the droplet, which continues to enlarge and elongate without breaking up. Note that the capillary numbers were defined based on the average inlet velocities, but encapsulation takes place at the orifice where the velocities are significantly high and comparable to velocities found in cross-channel flows. The success rate of encapsulation (η), defined as the ratio of the cell-laden droplets to the total generated droplets, decreases as both inner and outer capillary numbers increase.

## 3. Numerical Method

The governing equation can be expressed as a single set of Navier–Stokes equations, for all three phases, including cell, dispersed fluid, and continuous fluid. In this formulation, continuity and momentum equations can be written as:

(1) $$\begin{array}{ccc} \nabla \cdot u & = & 0, \end{array}$$

(2) $$\begin{array}{ccc} \frac{\partial \rho u}{\partial t}+\nabla \cdot \left(\rho \mathrm{uu}\right) & = & -\nabla p+\nabla \cdot \mu \left(\nabla u+\nabla u^{T}\right)+\nabla \cdot \tau +\int_{A}\sigma \kappa n\delta \left(x-x_{f}\right)dA, \end{array}$$

where μ, ρ, *p*, and u denote the viscosity, density, pressure, and velocity vector, respectively. The last term in the momentum equation is the interfacial tension acting on the surface in which σ, κ, and n are the interfacial tension coefficient, interface curvature, and unit vector normal to the interface, respectively. A delta function δ was used in the interfacial term, whose arguments x and $x_{f}$ are the points at which the equation is being evaluated and a point at the interface, respectively. Density and viscosity are constant in each of these three phases and vary discontinuously across the fluid interface, which can be expressed using an indicator function ϕ and density and viscosity at each of the three phases as follows:

(3) $$\begin{array}{c} \rho =\left\{\begin{array}{cc} \rho_{i}\phi +\rho_{o}\left(1-\phi \right) & \mathrm{if}\;\phi \leq 1\\ \rho_{c}\left(\phi -1\right)+\rho_{i}\left(2-\phi \right) & \mathrm{otherwise}, \end{array}\right.\\ \mu =\left\{\begin{array}{cc} \mu_{i}\phi +\mu_{o}\left(1-\phi \right) & \mathrm{if}\;\phi \leq 1\\ \mu_{c}\left(\phi -1\right)+\mu_{i}\left(2-\phi \right) & \mathrm{otherwise}, \end{array}\right. \end{array}$$

where *c*, *i*, and *o* denote the material properties of the cell, inner fluid, and outer fluid, respectively. The indicator function is updated after each time step to compute material properties accurately and is defined as:

(4) $$\begin{array}{c} \phi =\left\{\begin{array}{cc} 2 & \mathrm{in}\;\mathrm{the}\;\mathrm{cell},\\ 1 & \mathrm{in}\;\mathrm{the}\;\mathrm{encapsulating}\;\mathrm{droplet},\\ 0 & \mathrm{in}\;\mathrm{the}\;\mathrm{ambient}\;\mathrm{fluid}. \end{array}\right. \end{array}$$

The governing equations were discretized and solved in a single set of formulations using a front-tracking method. The front-tracking method used in this paper was validated using other experimental and numerical studies for different physics in multi-phase flows by Muradoglu et al. [34] and Tasoglu et al. [35]. The front-tracking method utilizes two separate grids: a staggered Eulerian grid to solve the flow field and a Lagrangian grid to track the interfaces between the fluids and also to compute the interfacial forces. The interfacial term then will be projected to the Eulerian grid to solve the Navier–Stokes equations. The Lagrangian grid, consisting of marker points and front elements, moves with the velocity of the flow field and is reconstructed by splitting/adding very large/very short elements after each time step. A complete description of the front-tracking method can be found in the work of Tryggvason et al. [36].

In this study, we used a uniform Cartesian grid for the Eulerian grid containing 128×1280 cells in the radial and axial directions, respectively. A grid convergence study showed that this grid was sufficient to reduce the spatial error below 2% for all flow configurations. Therefore, this grid resolution was used in all the results presented in this paper, unless specified otherwise.

## 4. Results and Discussion

In a flow-focusing technique for cell encapsulation, the inner and outer fluids flow in parallel streams. The interface between the two fluids moves forward, and in the orifice region, they focus to a narrow area, where stresses are built up and the two streams of flow speed up downstream of the flow. Viscous force, as well as the inertial and interfacial forces act on the interface, and the interplay between these forces develops a thread, which leads to pinch-off of the droplet. During the necking process, the cells that flow in the inner fluid may enter the forming droplet and produce cell-laden droplets. In a circular cross-section, within the orifice, the inner flow converges to a diameter equivalent to the cell size, leading and focusing the cells to the axis. The pattern of droplet formation highly depends on the flow-field conditions and geometry of the flow-focusing device.

The geometry of the orifice in the flow-focusing device plays an important role in focusing the flow and shaping the interface between fluids, which results in the production of cell-laden droplets. The annular geometry and the length and diameter of the orifice can significantly influence the pattern of droplet formation and cell encapsulation. Therefore, further simulations were conducted to investigate the effects of radius and length of the orifice and the nozzle-to-orifice distance on the droplet size, frequency of droplet generation, and probability of numbers of cells per droplet.

**Figure 3:**
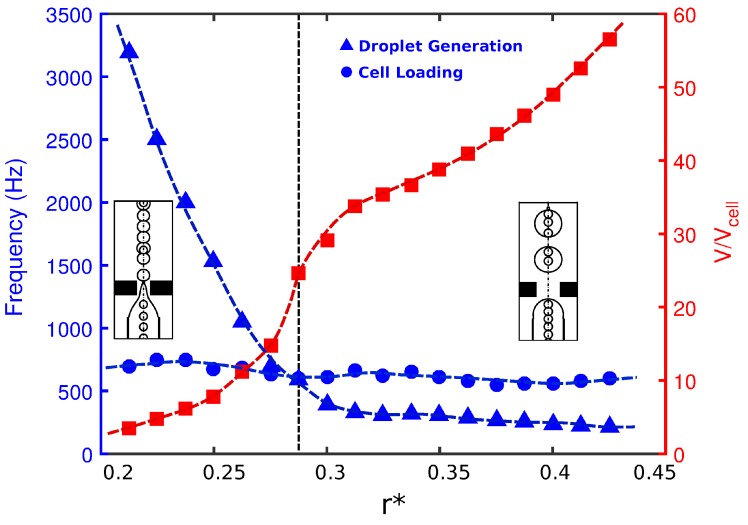
Frequency of droplet generation (triangles) versus frequency of cell loading (circles) and non-dimensional droplet size (squares) for configurations with different non-dimensional orifice radii at $Ca_{i}=Ca_{o}=10^{-3}$. Other geometrical parameters are kept fixed ($l^{*}=0.5$ and $d^{*}=0.75$).

The radius of the orifice can basically change the flow-field by reducing the cross-section of the orifice region. The effects of orifice radius $\left(r^{*}\right)$ on the droplet size, frequency of cell loading, and frequency of droplet generation are provided in Figure 3 for $Ca_{i}=Ca_{o}=10^{-3}$. The frequency of cell loading was approximately constant in all the simulations presented in this work. The loading frequency was essentially a measure of how fast the cells entered the orifice. The cells migrated with the inner fluid with a velocity, $V_{i}$. The inlet fluid velocity was fixed in the paper. Therefore, the cells moved with almost the same velocity as the inner fluid, and cell loading was almost constant. The throughput can be maximized by increasing the inner velocity. Any change in the width of the orifice will also change the velocity of the flow inside the orifice region. However, when the ligament was formed from the inner tube to the orifice (Figure 1c), the frequency of cell loading at the entrance of the orifice was dependent only on the inner fluid velocity. Thus, the effect of orifice width on cell loading frequency was negligible. The frequency of cell loading, i.e., the frequency at which the cells entered the orifice, was highly dependent on how fast they flowed in the orifice; however, the orifice geometry would be unable to manipulate it. Therefore, the frequency of cell loading for a fixed $Ca_{i}$ was roughly constant. As shown in Figure 3, for devices with a small orifice diameter, small droplets were generated at a frequency much higher than that for cell loading. Therefore, the cells had a short time to occupy the forming droplets. As a result, every single droplet encapsulated either a single cell or no cell at all (Mode II). By increasing the orifice radius, the droplet size increased, as also reported by Gupta et al. [31]. With the increase in orifice radius, the frequency of droplet generation decreased, and at a critical radius, it dropped below the frequency of cell loading. This indicated that the droplets were generated at a slower pace, increased in size, and included more than one cell per droplet.

**Figure 4:**
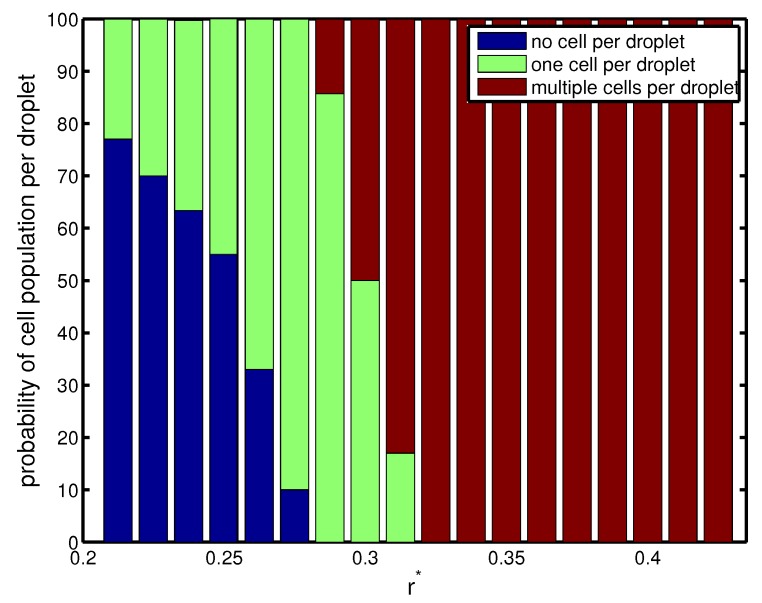
Probability of cell population per droplet (%) for configurations with different non-dimensional orifice radii at $Ca_{i}=Ca_{o}=10^{-3}$. Other geometrical parameters are kept fixed ($l^{*}=0.5$ and $d^{*}=0.75$).

The above phenomenon is corroborated in Figure 4, which depicts the probability of cell population per droplet in terms of orifice radius. For a narrow orifice $r^{*}≃0.21$, about 30% of droplets had cells, and 70% of them were empty. However, the probability increased with orifice radius. It was also seen that for a narrow radius of $0.25<r^{*}<0.3$, there was a 50–90% probability of capturing a single cell in a droplet, which was a desired criterion. At $r^{*}≃0.25$, the frequency of droplet generation was nearly equal to the frequency of cell loading. The cells entered the forming droplets at the same rate as the droplets were generated. Therefore, we had a more precise form of encapsulation, and almost 90% of droplets had exactly one cell inside them (Figure 4). Beyond this $r^{*}$, there was a higher probability of capturing multiple cells in the droplet since the droplet volume increased monotonically (Mode III).

**Figure 5:**
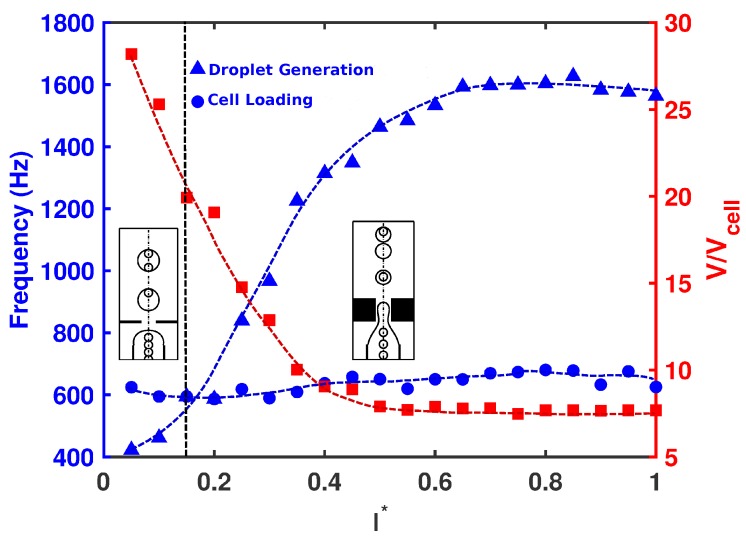
Frequency of droplet generation (triangles) versus frequency of cell loading (circles) and non-dimensional droplet size (squares) for configurations with different non-dimensional orifice lengths at $Ca_{i}=Ca_{o}=10^{-3}$. Other geometrical parameters are kept fixed ($r^{*}=0.25$ and $d^{*}=0.75$).

**Figure 6:**
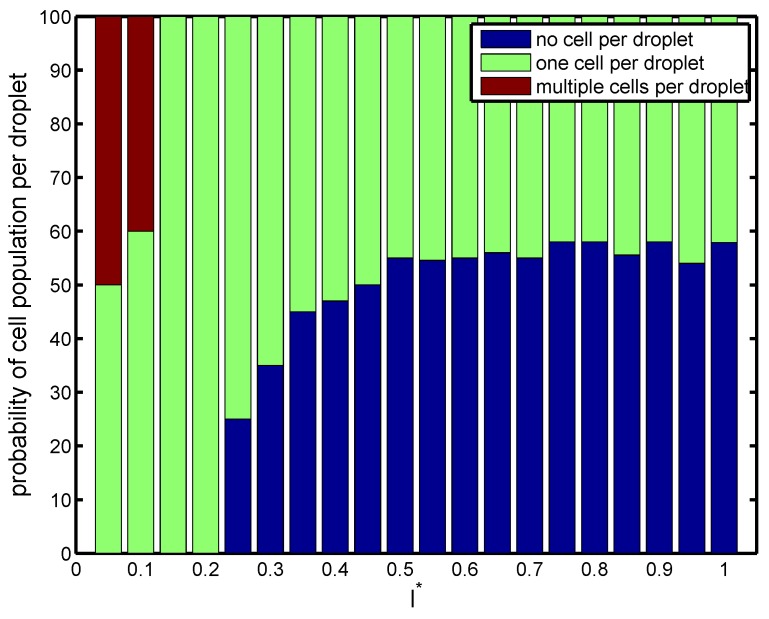
Probability of cell population per droplet (%) for configurations with different non-dimensional orifice lengths at $Ca_{i}=Ca_{o}=10^{-3}$. Other geometrical parameters are kept fixed ($r^{*}=0.25$ and $d^{*}=0.75$).

Next, the effect of orifice length $\left(l^{*}\right)$ was investigated on the droplet size, frequency of cell loading, frequency of droplet generation, and cell population per droplet at $Ca_{i}=Ca_{o}=10^{-3}$. The radius of the orifice and nozzle-to-orifice distance were kept fixed ($r^{*}=0.25$ and $d^{*}=0.75$). As seen in Figure 5, short orifices produced large droplets downstream at low frequency. This allowed the cells to migrate fast enough to fill the forming droplet. All the droplets carried cells, and there was a high possibility of having multiple cells inside the droplet (Mode III). As the length of the orifice increased, the droplets decreased in size and the frequency of droplet generation increased. Beyond a critical value of $l^{*}≃0.15$, the frequency of droplet generation overcame that of cell loading, and the droplet production happened faster than the cells encapsulated in forming droplets. The droplets were generated fast and did not allow multiple cells to enter the forming droplets. As a result, at most one cell could be encapsulated by each droplet, and single cell encapsulation occurred (Mode II). As shown in Figure 6, for all orifice lengths, the probability of single cell encapsulation was greater than 40%. For devices with $l^{*}=0.15$ and 0.2, cells entered the forming droplet almost at the same rate as that of droplet generation, and therefore, 100% of the droplets had a single cell, which was the ideal case. As the orifice length increased to nearly twice the radius $\left(l^{*}\geq 0.5\right)$, the probability decreased with more than half the droplets remaining empty. For all orifice lengths above $l^{*}>0.5$, cell encapsulation remained insensitive to orifice length, and the drop size and rate of generation remained more or less constant. For a short orifice, the pinch-off occurred after the junction, whereas for a longer orifice, the pinch-off occurred within the orifice. Beyond $l^{*}≃0.5$, regardless of the orifice length, the droplet always formed at the same place within the orifice. After the droplet was formed within the orifice, the droplet volume remained constant. It was also observed by Gupta et al. [31] that increasing orifice length first decreased the drop size, and after a critical value of $l^{*}$, the drop size was insensitive to a further increase in orifice length.

**Figure 7:**
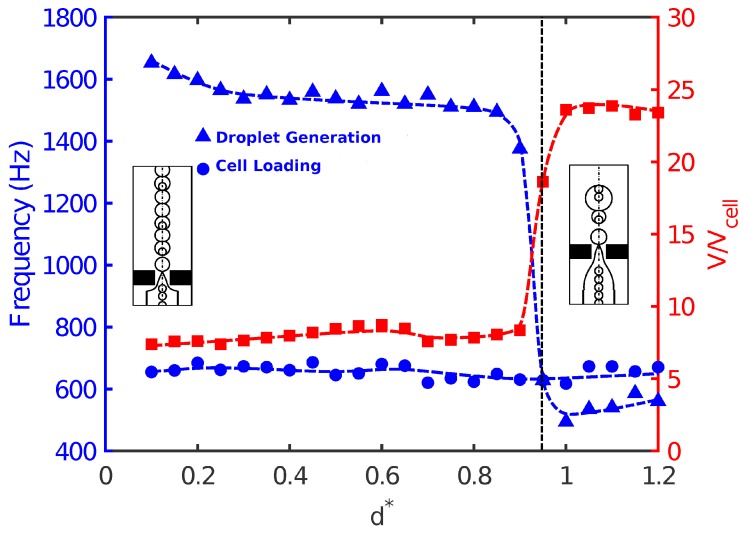
Frequency of droplet generation (triangles) versus frequency of cell loading (circles) and non-dimensional droplet size (squares) for configurations with different non-dimensional nozzle-to-orifice distances at $Ca_{i}=Ca_{o}=10^{-3}$. Other geometrical parameters are kept fixed ($r^{*}=0.25$ and $l^{*}=0.5$).

**Figure 8:**
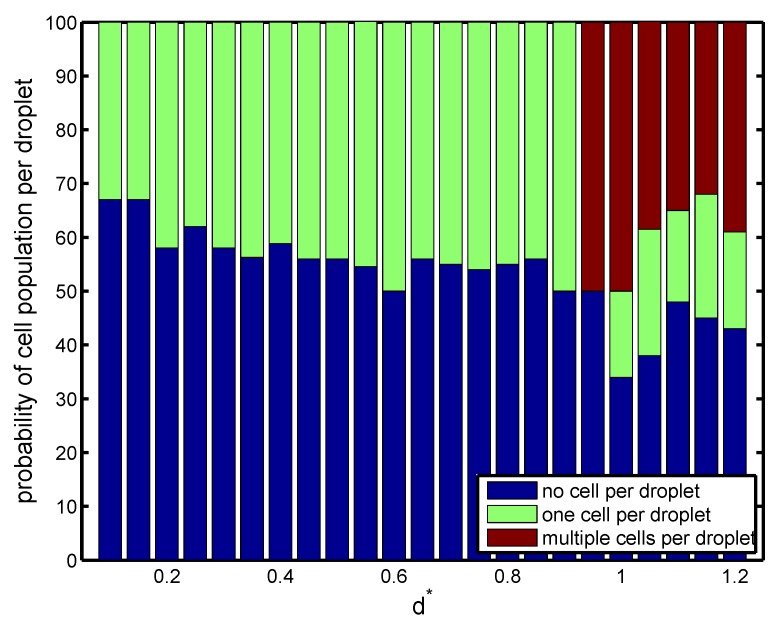
Probability of cell population per droplet (%) for configurations with different non-dimensional nozzle-to-orifice distances at $Ca_{i}=Ca_{o}=10^{-3}$. Other geometrical parameters are kept fixed ($r^{*}=0.25$ and $l^{*}=0.5$).

Finally, the effect of nozzle-to-orifice distance $\left(d^{*}\right)$ on droplet size, frequency of droplet generation, and population of cells was studied for $Ca_{i}=Ca_{o}=10^{-3}$. When the orifice was very close to the nozzle, the evolution of fluid interface tended to be fast due to the short distance between the nozzle and orifice. The flow focused rapidly and in a more controlled manner, and small high-throughput droplets were produced in the orifice region. The droplets were uniform in size, and the frequency of droplet generation was higher than that of cell loading. Therefore, single cell encapsulation occurred, and the success rate was low in Mode II. As the distance between the nozzle and orifice increased, the droplet size slightly decreased with a corresponding increase in the frequency of droplet generation. However, a more noticeable effect of nozzle-to-orifice distance on cell encapsulation was observed near $d^{*}≃1.0$. Figure 7 shows that slightly before $d^{*}=1.0$, a sudden jump in drop size and droplet generation frequency occurred. Droplets were enlarged almost three times with a significant decrease in droplet generation. This was because the large distance between the nozzle and the orifice did not allow a smooth and controllable focusing of the flow. As a result, non-uniform droplets were generated with a random pattern. This was not a desirable situation, since it would be difficult to encapsulate uniformly. There was a random pattern of empty droplets, droplets with a single cell, and droplets with multiple cells (Figure 8). Such an increase in droplet size was also seen by Gupta et al. [31] in their 2D numerical study in the flow-focusing configuration.

**Figure 9:**
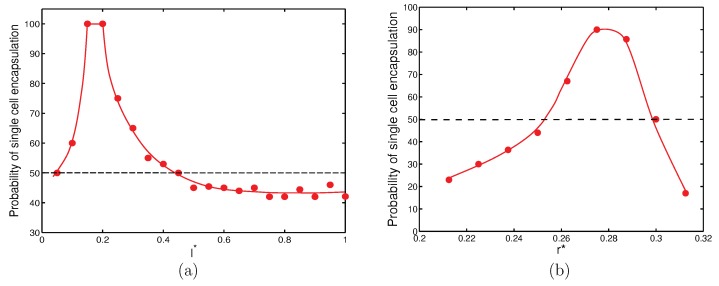
Probability of single cell encapsulation (%) for configurations at $Ca_{i}=Ca_{o}=10^{-3}$ with different non-dimensional (**a**) orifice length at fixed $r^{*}=0.25$, $d^{*}=0.75$ and (**b**) orifice radius at fixed $l^{*}=0.5$, $d^{*}=0.75$.

In summary, success in encapsulation of single cells can be achieved by judiciously selecting the orifice length and orifice radius. With the nozzle-to-orifice distance fixed ($l^{*}=0.75$), it was possible to develop plots for appropriate orifice length and orifice radius for which there was a high possibility of encapsulating single cells consistently (Figure 9). Figure 9a shows that for $r^{*}=0.25$, there was a non-dimensional length, $l^{*}=0.15$ and 0.2, at which there was a 100% probability of capturing a single cell in a droplet. It can also be seen that if $l^{*}$ was between 0.05 and 0.45, there was at least 50% probability of capturing single cells in droplets. Figure 9b shows that for $l^{*}=0.5$, a high probability of single cell encapsulation can be achieved for $0.25<r^{*}<0.3$.

## 5. Conclusions

A parametric study was performed to investigate the influence of flow-focusing geometry on encapsulation of living cells. A three-phase front-tracking computational study was conducted to simulate single and multiple cell encapsulation. Single cell encapsulation, i.e., having at most one cell per droplet, occurred when the capillary numbers of the inner and outer fluids were of the same order of magnitude. The effects of three main geometrical characteristics including the orifice radius, orifice length, and nozzle-to-orifice distance were studied for $Ca_{i}=Ca_{o}=10^{-3}$. The simulations suggested that short orifices can help reach a uniform high-throughput production of droplets. In general, an orifice with a radius equal to their length, but one-fourth that of the outer tube radius and kept at least three radii away from the inner tube exit produced the best result of encapsulating at least one cell per droplet at a high probability. As the orifice radius increased, the drop size and number of cells per droplet increased. Short orifices produced large drops with multiple cells per droplet. As the length of orifice increased, the frequency of droplet production increased, and small droplets were generated more consistently with at most one cell per droplet. Beyond $l^{*}≃0.5$, cell encapsulation appeared to be insensitive to orifice length. The distance between the nozzle and orifice was another parameter for controlling cell encapsulation in parallel flow-focusing geometries. A short nozzle-to-orifice distance resulted in controlled droplet production, while a long distance affected flow-focusing unfavorably in the vicinity of the orifice, making the droplet generation unpredictable and non-uniform.

In general, it was found that there was a high probability of single cell encapsulation for $r^{*}≃0.25$ and $d^{*}≃0.75$ and $0.05\leq l^{*}\leq 0.45$. In other words, for best results, the orifice needed to be placed at three times the orifice radius from the inner tube exit. The orifice should also have a radius one-fourth of the outer tube radius, with a length $0.05\leq l^{*}\leq 0.45$.
